# Pattern Visually Evoked Potentials in Japanese Girl With Optic Neuritis and Seropositive to Anti-myelin Oligodendrocyte Glycoprotein (MOG) Antibody

**DOI:** 10.3389/fneur.2019.01339

**Published:** 2019-12-19

**Authors:** Shunichiro Takano, Aya Hanabusa, Yuji Yoshikawa, Kaori Sassa, Airi Shimura, Takuhei Shoji, Hisao Ohde, Kei Shinoda, Hideo Yamanouchi

**Affiliations:** ^1^Department of Ophthalmology, Faculty of Medicine, Saitama Medical University, Saitama, Japan; ^2^Departments of Pediatrics, Comprehensive Epilepsy Center, Faculty of Medicine, Saitama Medical University, Saitama, Japan; ^3^Makuhari Ode Eye Clinic, Chiba, Japan

**Keywords:** optic neuritis, myelin-oligodendrocyte glycoprotein (MOG), anti-MOG antibody, aquaporin 4 (AQP4), anti-AQP4 antibody, pattern visual-evoked potentials

## Abstract

**Purpose:** To describe a Japanese girl with unilateral optic neuritis who was seropositive for the anti-myelin-oligodendrocyte glycoprotein (MOG). Serial recordings of the pattern visual evoked potentials (pVEPs) were made to follow the dynamic changes of the disease activity.

**Observations:** A 5-year-old girl developed a sudden reduction of vision and deep ocular pain in her right eye. On examination at our university hospital, the best-corrected visual acuity (BCVA) was light perception, and a swelling of the optic disc and tortuous vessels at the posterior pole of the right eye were observed. MRI demonstrated that her right optic nerve was hyperintense on short TI inversion recovery (STIR) sequence. A diagnosis of right papillitis was made, and she was treated with steroid pulse therapy followed by a gradual tapering of oral prednisolone. The visual acuity decreased to no light perception and plasmapheresis combined with high-dose intravenous immunoglobulin therapy was performed. The decimal visual acuity rapidly improved and recovered to 1.2, and no recurrence was observed for at least 1 year. On day 19, she was found to be anti-MOG antibody positive and anti-Aquaporin 4 antibody negative. pVEPs were recorded during the course of the disease process which showed the dynamic changes of the physiology of the visual pathways. The implicit times of the N75 and P100 components were prolonged in the right eye in the acute phase. The right visual acuity remained at 1.2 for at least 1 year, but the implicit times of the N75 and P100 components of the pVEPs of the right eye were still prolonged compared to left eye.

**Conclusion:** Our findings indicate a positive relationship between the anti-MOG antibodies-positivity and the prolonged pVEPs. Further analyses of the pVEPs and other clinical findings of the optic neuritis are needed to establish the clinical significance of the anti-MOG antibodies positivity and optic neuritis for the diagnosis, treatment, and prognosis for this disease.

## Background

Myelin-oligodendrocyte glycoprotein (MOG) is a surface protein located on the oligodendrocytes of the central nervous system (CNS) and the optic nerves (1, 2). Autoantibodies against MOG are associated with acute disseminated encephalomyelitis (ADEM) in children and the opticospinal type of multiple sclerosis (MS) in adults (3–5). In addition, anti-MOG antibodies are frequently detected in patients with recurrent optic neuritis at ages ≤ 18-years (6). The optic neuritis can be a part of ADEM, MS, and the neuromyelitis optica spectrum disorders (NMOSDs). Although anti-aquaporin 4 (AQP4) antibody-positivity is a key in the diagnosis of NMOSD, anti-MOG antibodies have played an important role in NMO because a proportion of anti-AQP4 antibody-negative cases have anti-MOG antibody positivity (7, 8). Kim et al. reported that the predominant demyelinating disease found in anti-MOG antibody-positive patients was optic neuritis (83%) (3). Because patients with anti-MOG antibody may show a relapsing-remitting disease course, and MRI evidence shows a dissemination in space and time, anti-MOG antibody positive optic neuritis might have been diagnosed as optic neuritis associated with multiple sclerosis (9–11).

Although the characteristics of the visual evoked potentials (VEPs) are not used to diagnose MS, they have been helpful for the diagnosis and monitoring of the optic neuritis in patients with MS. The prolongation of the P100 implicit times is accepted as a pathognomonic sign of optic nerve demyelination in MS (12–15).

We present our findings in a young Japanese girl who developed acute optic neuritis and was anti-MOG antibody-positive. Pattern visually evoked potentials were recorded during the recovery course.

## Case Presentation

**Figure 1:**
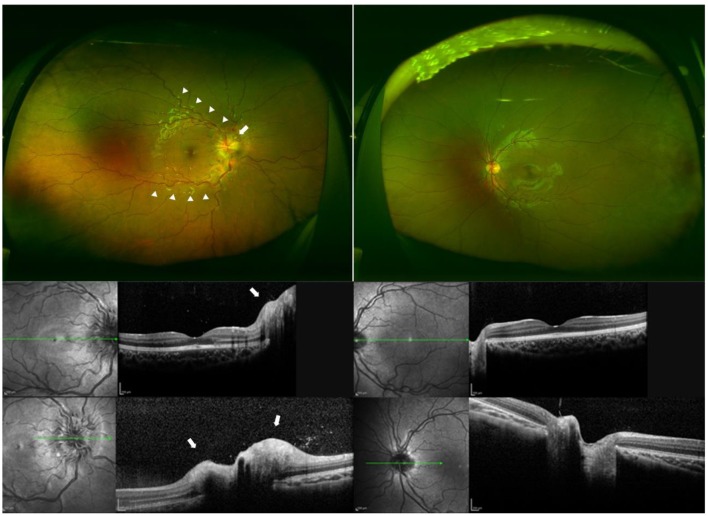
Fundus photographs and optical coherence tomographic (OCT) images of a patient with unilateral optic neuritis and seropositivity to anti-myelin oligodendrocyte glycoprotein (MOG) antibody. **(Top)** Ultra-widefield fundus photographs of each eye at the initial visit showing tortuous arcade vessels (arrowheads) and optic disc swelling (arrow) in the right eye. The decimal best-corrected visual acuity (BCVA) was light perception in the right eye and 1.2 in the left eye. **(Middle)** Optical coherence tomographic (OCT) images of the posterior pole of each eye at the initial visit showing optic disc swelling in the right eye (arrow). Left, right eye; right, left eye. **(Bottom)** OCT images of the optic disc of each eye at the initial visit showing optic disc swelling in the right eye (arrow). Left, right eye; right, left eye.

**Figure 2:**
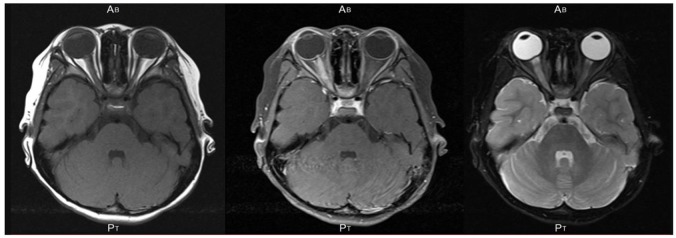
An axial section of magnetic resonance images of the brain and orbits in the patient showing hyperintensity of the right optic nerve on short TI inversion recovery (STIR) sequence **(Left)**, T1-weighted image with gadolinium enhancement **(Middle)**, and T2-weighted image **(Right)**. No cerebral lesion was detected. An axial section of MRI of the brain and orbits in the patient showing hyperintensity of the right optic nerve on short TI inversion recovery (STIR) sequence and no cerebral lesion.

A 5-year-old girl developed a sudden reduction in her vision and had deep ocular pain in her right eye. She visited a private eye clinic on the following day. She had no noteworthy medical and family histories. Her decimal visual acuity (VA) was 0.7 OD and 1.2 OS with a relative afferent pupillary defect in the right eye on day 2. She was referred to the Saitama Medical University Hospital, and ophthalmoscopy and optical coherence tomography showed a swelling of the optic disc and tortuous vessels at posterior pole of the right eye on day 3 (Figure 1). Neurological and general examinations were within normal limits. MRI demonstrated hyperintensity of her right optic nerve on short TI inversion recovery (STIR) sequence and no cerebral lesions (Figure 2). On the initial visit to Saitama Medical University, patient's blood was drawn for laboratory examinations and for serum antibodies against AQP4 and MOG. Laboratory examinations showed that the blood and cerebrospinal fluid tests were within the normal limits except a few items (Supplemental Table 1). Spinal MRI showed no abnormalities in the cervical, thoracic, and lumbar spinal cord.

Ischemic optic neuropathy, traumatic neuropathy, toxic-nutritional optic neuropathy, rhinogenic optic neuritis, and hereditary optic neuropathy were considered as a differential diagnosis and were denied from various clinical findings. She was diagnosed with right papillitis and treated with 450 mg of intravenous methylprednisolone pulses for 3 days (days 3–5) followed by a gradual tapering of oral prednisolone (Table 1). On day 5, the best-corrected visual acuity (BCVA) decreased to no light perception, and plasmapheresis was performed for 3 days (days 6, 9, and 12) combined with high dose intravenous immunoglobulin therapy (160 mg/kg, total 2.5 g) for 1 day (day 10) (Table 1). Because the patient's vision loss was so severe, we started systemic treatment to prevent a relapse after discussion with patient's mother. The decimal visual acuity rapidly improved and reached 1.0 on day 13 [Table 1; (16)]. The swelling of the optic disc and tortuosity of the retinal vessel of the right eye disappeared on days 35 and 63, respectively. From the examination of the blood drawn on the initial visit, it turned out on day 9 that she was anti-AQP4 antibody-negative and on day 19 that anti-MOG antibody-positive.

**Table 1:**
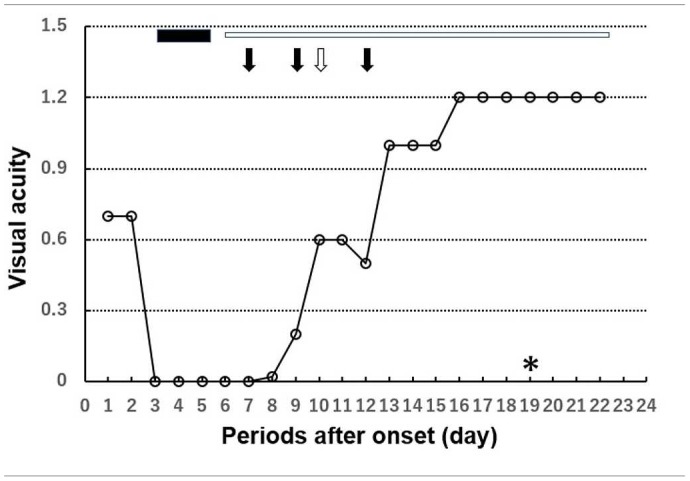
Clinical course with visual acuity and treatments.

Visual acuities of “light perception” and “no light perception” were assigned values of 2.8 and 2.9 logMAR units, respectively (16). The black bar shows steroid pulse therapy for 3 days (450 mg of intravenous methylprednisolone pulses on days 3–5). The white bar shows steroid maintenance therapy. The black arrow shows plasmapheresis therapy (days 6, 9, and 12). The white arrow shows high-dose intravenous immunoglobulin therapy (160 mg/kg, total 2.5 g on day 10). *On day 19, it turned out that anti-MOG antibody positive.

**Figure 3:**
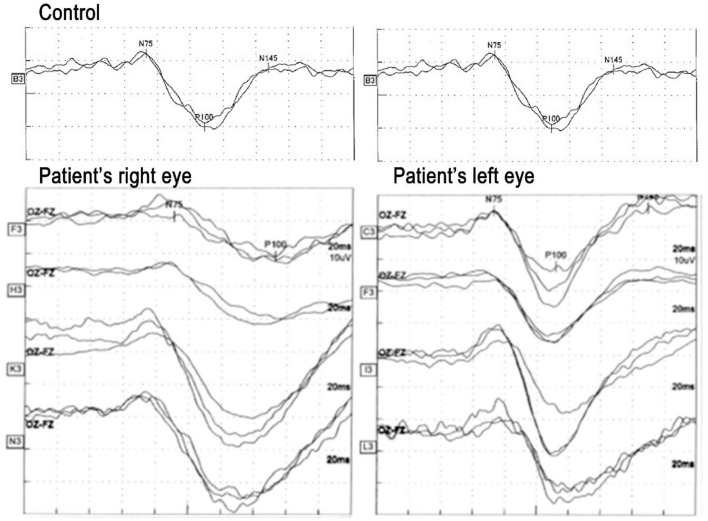
Pattern visual evoked potentials (pVEPs) recorded several times during the experimental period. Left pVEPs elicited by stimulating the right eye and that elicited by stimulating the left eye. Top pVEPs recorded on day 14 when visual acuity was 1.0, second row: on day 21 when visual acuity was 1.2, third row: on day 109 when visual acuity was 1.2, and bottom: on day 305 when visual acuity was 1.2. The implicit time of N75 was prolonged until day 21 in the right eye compared to the left eye, and thereafter it seems to normalize and stable for almost 1 year. On the other hand, the implicit time of the P100 component was prolonged in the right eye compared to the left eye throughout of the following period. The implicit time in the right eye became shorter with time and stabilized between the day 109 and 305 while the implicit time in the left eye was stable during entire period. An example from normal control is shown in the top.

Pattern VEPs (pVEPs) were recorded several times during the course of the disease with the recording parameters conforming to the International Society of Clinical Electrophysiology of Vision (ISCEV) standards except that the checkerboard size was ~2° (17). The findings represented the dynamic changes in the physiology of the visual pathways objectively (Figure 3; Table 2). The implicit times of the N75 and P100 components of the pVEPs of the right eye were prolonged compared to that of the normal fellow eye when the VEPs were elicited by stimulating the right eye throughout the follow-up period. The implicit times of the right eye became shorter on day 109 and remained prolonged on day 316.

**Table 2:** The latency of N75 and P100 components in pattern visual evoked potentials.

**Periods from onset (days)**	**Visual acuity**		**Latency (msec)**			
			**N75**		**P100**	
	**Rt**	**Lt**	**Rt**	**Lt**	**Rt**	**Lt**
14	1.0	1.2	90.8	72.8	152.8	111.8
21	1.2	1.2	85.8	63.8	145.6	108.8
109	1.2	1.2	75.4	75.4	130.8	111.8
305	1.2	1.2	73.4	76.8	132	118.8

Although the patient reported that she felt that the image of the right eye was relative dark compared to the left eye, the decimal BCVA of the right eye has remained at 1.2 for at least 1 year. The implicit time of N75 was prolonged in the right eye compared to the left eye until day 21, and thereafter it seemed to normalize and become stable for almost 1 year. On the other hand, the implicit time of the P100 component was prolonged in the right eye compared to the left eye throughout the follow-up period.

Five milligram of oral prednisolone has been used every other day, and no recurrences have been observed for at least 1 year.

## Discussion

Anti-MOG antibodies have been recognized to be specific biomarkers for different disease entities, such as anti-AQP4 antibodies for NMO, but their pathogenic role has not been fully determined (2–4, 11). The diseases associated with MOG antibody are now recognized as a distinct nosological entity with specific management and therapeutic requirements (18). Because patients with optic neuritis often have a severe reduction of visual acuity that develops in a short time, a rapid decision on the therapeutic strategy is required before seropositivity or negativity for anti-MOG and anti-AQP4 antibodies becomes clear. Several clinical features that are useful for the diagnosis have been reported, and the pVEP findings are very important especially in children (15, 18–20).

The prolongation of the implicit times of the pVEPs represents the neurotransmission rate, and its delay is a good biomarker to evaluate functional damage of the demyelinated optic neuritis. In our case, the prolongation was present in spite of the recovery of the fundus appearance and the BCVA. The prolongation stabilized in the chronic phase and did not return to that of the healthy fellow eye. Our findings are from single case and cannot be expanded for other optic neuritis. It has been reported that there were no significant differences in the incidence of eyes with prolonged P100 implicit time between the anti-MOG antibody-positive patients and anti-AQP4 antibody-positive patients; i.e., 57% of anti-MOG antibody-positive patients and 50% of anti-AQP4 antibody-positive patients had prolonged implicit times (15). A more accurate comparison such as in the degree of the implicit time delay between the two groups might differentiate patients with optic neuritis and anti-MOG antibody-positivity from other optic neuritis. Further investigations on the pVEPs for the possibility of differentiating anti-MOG antibody-positive optic neuritis from anti-AQP4 antibody-positive optic neuritis will be important. In addition, the use of the implicit times as an indicator of the effectiveness of a new therapeutic regimen should be examined.

We began our treatment with steroid pulse therapy using the same regimen used to treat anti-AQP4 antibody-positive optic neuritis followed by post-therapy administration of oral steroids plus immunotherapy. Because systematic treatment to prevent a relapse of anti-MOG positive pathologies is not well-accepted, we, the pediatrician and ophthalmologist, discussed this treatment with the patient's mother. Because this was prior to the detection of the sero-positivity or negativity of anti-MOG antibody and anti-AQP4 antibody and the patient's vision loss was so severe, we started systemic treatment to try to prevent a relapse. Anti-MOG antibody-positive optic neuritis and anti-AQP4 antibody-positive optic neuritis have different clinical features and different optic nerve damage. Thus, different therapeutic strategies needed to be determined (15, 18–20). The determinations of the clinical characteristics and analyses of the pVEPs of these different clinical entities should be helpful in the understanding of the pathology and establishing the best therapeutic regimen (11, 21). Our case had persistent VEP abnormalities despite clinical recovery in the right eye. Jarius et al. reported that some anti-MOG antibody-positive patients with only transverse myelitis and no optic neuritis had delays in the implicit times of P100 of the pVEP This suggested a subclinical optic nerve dysfunction (11). Further studies are necessary to determine the implications of the prolonged P100 implicit time as to whether it is only a sign of old optic neuritis, a sign of relapse, or an indicative marker for the anti-MOG antibody activity. In conclusion, we present our findings in a 5-year-old Japanese patient with unilateral optic neuritis and seropositive to anti-MOG antibodies. Steroid pulse therapy followed by post-therapy administration of oral steroids plus immunotherapy led to a recovery of the appearance of the fundus. However, the implicit times of the pVEPs remained longer than that of the normal fellow eye. Additional case reports and further analyses of the pVEPs should provide new insights into anti-MOG antibodies-related diseases and whether the implicit time of the pVEPs can be a marker for eyes with subclinical unilateral optic neuritis and seropositive to anti-MOG antibodies.

## Data Availability Statement

All datasets generated for this study are included in the article/Supplementary Material.

## Ethics Statement

Written informed consent to publish this case report and any accommodating data and images was obtained from the patient's next of kin.

## Author Contributions

AH, KSa, HY, and KSh cared for the patient, including during assessment and treatment throughout and in the follow up period. ST, YY, AS, TS, and KSh collected data, analyzed the ophthalmological findings, and gave critical suggestions. ST, AH, and KSh prepared figures. KSh prepared a draft. ST, YY, TS, HO, and HY revised and finalized it. All authors agree to be accountable for all aspects of work. All authors attest that they meet the current ICMJE criteria for authorship.

### Conflict of Interest

The authors declare that the research was conducted in the absence of any commercial or financial relationships that could be construed as a potential conflict of interest.
